# Muscle Proteomic and Transcriptomic Profiling of Healthy Aging and Metabolic Syndrome in Men

**DOI:** 10.3390/ijms22084205

**Published:** 2021-04-19

**Authors:** Marine Gueugneau, Cécile Coudy-Gandilhon, Christophe Chambon, Julien Verney, Daniel Taillandier, Lydie Combaret, Cécile Polge, Stéphane Walrand, Frédéric Roche, Jean-Claude Barthélémy, Léonard Féasson, Daniel Béchet

**Affiliations:** 1 Université Clermont Auvergne, INRAE, UNH, Unité de Nutrition Humaine, CRNH Auvergne, 63000 Clermont-Ferrand, France; marine.gueugneau@inrae.fr (M.G.); cecile.coudy-gandilhon@inrae.fr (C.C.-G.); daniel.taillandier@inrae.fr (D.T.); lydie.combaret@inrae.fr (L.C.); cecile.polge@inrae.fr (C.P.); stephane.walrand@inrae.fr (S.W.); 2 Metabolomic and Proteomic Exploration Facility, Université Clermont Auvergne, INRAE, 63000 Clermont-Ferrand, France; christophe.chambon@inrae.fr; 3 Laboratoire AME2P, Université Clermont Auvergne, 3533 Clermont-Ferrand, France; julien.verney@uca.fr; 4 Service de Physiologie Clinique et de l’Exercice, CHU Saint Etienne, 42055 Saint Etienne, France; frederic.roche@chu-st-etienne.fr (F.R.); jc.barthelemy@univ-st-etienne.fr (J.-C.B.); 5 INSERM, SAINBIOSE, U1059, Dysfonction Vasculaire et Hémostase, Université Jean-Monnet, 42055 Saint-Etienne, France; 6 Unité de Myologie, Service de Physiologie Clinique et de l’Exercice, Centre Référent Maladies Neuromusculaires Euro-NmD, 42000 CHU de Saint-Etienne, France; leonard.feasson@univ-st-etienne.fr; 7 Laboratoire Interuniversitaire de Biologie de la Motricité, Université de Lyon, Université Jean Monnet Saint-Etienne, 69000 Lyon, France

**Keywords:** skeletal muscle, aging, sarcopenia, metabolic syndrome, proteome, transcriptome

## Abstract

(1) Background: Aging is associated with a progressive decline in muscle mass and function. Aging is also a primary risk factor for metabolic syndrome, which further alters muscle metabolism. However, the molecular mechanisms involved remain to be clarified. Herein we performed omic profiling to decipher in muscle which dominating processes are associated with healthy aging and metabolic syndrome in old men. (2) Methods: This study included 15 healthy young, 15 healthy old, and 9 old men with metabolic syndrome. Old men were selected from a well-characterized cohort, and each vastus lateralis biopsy was used to combine global transcriptomic and proteomic analyses. (3) Results: Over-representation analysis of differentially expressed genes (ORA) and functional class scoring of pathways (FCS) indicated that healthy aging was mainly associated with upregulations of apoptosis and immune function and downregulations of glycolysis and protein catabolism. ORA and FCS indicated that with metabolic syndrome the dominating biological processes were upregulation of proteolysis and downregulation of oxidative phosphorylation. Proteomic profiling matched 586 muscle proteins between individuals. The proteome of healthy aging revealed modifications consistent with a fast-to-slow transition and downregulation of glycolysis. These transitions were reduced with metabolic syndrome, which was more associated with alterations in NADH/NAD^+^ shuttle and β-oxidation. Proteomic profiling further showed that all old muscles overexpressed protein chaperones to preserve proteostasis and myofiber integrity. There was also evidence of aging-related increases in reactive oxygen species but better detoxifications of cytotoxic aldehydes and membrane protection in healthy than in metabolic syndrome muscles. (4) Conclusions: Most candidate proteins and mRNAs identified herein constitute putative muscle biomarkers of healthy aging and metabolic syndrome in old men.

## 1. Introduction

Aging affects most tissues and physiologic functions, but one of the most affected organs is the skeletal muscle. Between the ages of 20 and 80 years, the cross-sectional area of the vastus lateralis muscle may be reduced by up to 40% [1,2]. This progressive decline in muscle mass and function (referred to as sarcopenia) contributes to both loss of autonomy and increased prevalence for frailty. Noteworthy, independent of other risk factors or diseases, a low skeletal muscle mass or strength is reported to be a predictor of morbidity [3] and mortality [4].

Healthy skeletal muscles are central not only for coordinated movements and postural control but also for general well-being. Hence, age-related loss in skeletal muscle contractile strength increases the risk of impaired mobility, falls, and loss of autonomy. Skeletal muscle, which is the most abundant tissue in the adult body, further plays a central role as a reserve for energy and amino acids and is a major site for fatty acid oxidation, carbohydrate metabolism, and heat homeostasis [5]. Hence, age-related loss of muscle mass also triggers severe metabolic side effects, including metabolic syndrome and frailty in the elderly. Aging is indeed a primary risk factor for metabolic syndrome, a cluster of metabolic and cardiovascular symptoms that increase the risk for type 2 diabetes, cardiovascular diseases, and mortality from all causes. A large survey of the U.S. population in fact pointed out that metabolic syndrome prevalence strongly increases with advancing age [6].

Numerous theories have been proposed to explain sarcopenia. Obviously, muscle aging is a multifactorial phenomenon, which implicates intrinsic factors such as perturbations in the endocrine system (somatopause, menopause, andropause, adrenopause) [7] and oxylipins [8], increase in proinflammatory cytokines with attendant chronic inflammation (referred to as inflamm-aging) [9], motor units denervation–reinnervation [10,11], decreased muscle regeneration capacity [12,13], and altered protein turnover [14]. Undoubtedly, extrinsic factors such as diet and exercise further play important roles [15].

At the cellular level, chronological aging is characterized by a loss of muscle fibers and by type II fiber atrophy [1]. Several studies have also reported fiber deformation [16,17], an increased proportion of non-contractile (adipose and connective) tissues within muscles [18], fibrosis of the extracellular matrix (ECM) [19,20,21], and altered microvascularization in the aged skeletal muscle [22,23]. Perturbations in mitochondria have also been noted in aging, including reduced [24] or not [25,26] oxidative capacity and increased production of reactive oxygen species. Moreover, skeletal muscle is a major site of fatty acid oxidation and dysregulations of lipid metabolism, with accumulation or delocalization of intramyocellular lipid droplets occurring in the old muscle [17,27,28].

The overall functional, structural, and biochemical alterations of the skeletal muscle have been extensively studied for chronological aging, but the molecular mechanisms implicated remain to be specified. At the molecular level, whole-genome expression profiling [29,30,31], together with meta-analysis of microarray experiments [32], have been used to identify genes that change expression with chronological age in the human skeletal muscle. The differential expression profiles of mRNAs constitute a first essential level of information, but analyses of the expression profile of proteins in aging are also required. Several proteomic studies investigated muscle aging in rodents [33,34,35,36]; however, few were conducted with human muscle [24,37,38,39], and surprisingly, none assessed age-prevalent pathologies such as metabolic syndrome. Moreover, no previous study combined immunohistology and omics investigations to provide an integrative view of muscle aging in humans. 

PROOF (PROgnostic indicator OF cardiovascular and cerebrovascular events) is a unique cohort of elderly subjects perfectly well characterized since 2001 for many clinical, behavioral, and biological criteria [40]. Old men were previously selected from PROOF to describe age-dependent and metabolic-syndrome-associated alterations in muscle fiber morphometry, lipid droplets, oxidative metabolism, capillarization, and ECM fibrosis [17,22]. Using the same muscle biopsies, we performed transcriptome and proteome profiling to decipher the dominating processes associated with healthy aging and metabolic syndrome in old men.

## 2. Results and Discussion

### 2.1. Subject Clinical Characteristics

Our study included three groups of men: healthy young (YO), healthy old (EL), and old men with metabolic syndrome (SX). Subjects had not been participating in any program of resistance or endurance exercise for the previous 12 mo. As a rise in blood pressure is a normal part of aging [41], the EL group reflected a representative elderly population with 50% pre-hypertension (130/85 mm Hg < blood pressure < 140/90 mm Hg). Table 1 provides the characteristics of the subjects involved in the present study. Body weight and body mass index were similar for YO and EL but elevated for SX men. VO_2peak_ decreased and blood pressure increased with aging and in response to metabolic syndrome. There was also an age-related decline in muscle specific strength. Daily energy expenditure was similar between EL and SX groups. Waist circumference and fasting blood glucose and triglyceride concentrations were significantly higher for SX when compared with EL. DEXA analyses showed that SX men had a higher percentage of body fat mass and a lower percentage of appendicular lean mass when compared with EL men.

### 2.2. Transcriptomic Profiling of Muscle Aging and Comparison with Other Databases

Whole genome expression profiling indicated that 479 genes were differentially expressed between YO and old (EL and/or SX) muscles (Table S1). Previous transcriptome databases established for human muscle aging [29,30,31,32,42,43,44,45,46] sharply differed with regard to age, gender, or health status. We nonetheless tested the significance of the overlap between these databases and our list to eventually identify a common signature, despite major health divergences. Among our 479 genes, 241 have been reported in previous transcriptomic databases (all comparisons are shown in Table S2). This analysis indicates that, despite diverse clinical backgrounds, the genes most commonly associated with aging were related to muscle regeneration (CDKN1A/p21, HOXB2, DMRT2), neurite outgrowth (LGI1, SCN4B, FEZ2), stress signaling (ATP1B4, GADD45G, C21orf7), angiogenesis (KLF5, CRIM1), and energy metabolism (NT5C2, PPARGC1A/PGC1α).

The PROOF cohort used in the present study is a unique cohort of elderly subjects perfectly well characterized since 2001 for more than 200 clinical, behavioral, and biological criteria, including cardiac and cerebro-vascular diseases, lung diseases, metabolic disorders, sleepiness, autonomic nervous system status, and medications [40]. This cohort therefore enabled an optimal analysis of healthy aging and of aging associated with metabolic syndrome.

### 2.3. Transcriptomic Analysis of Muscle Healthy Aging in Men

**Figure 1:**
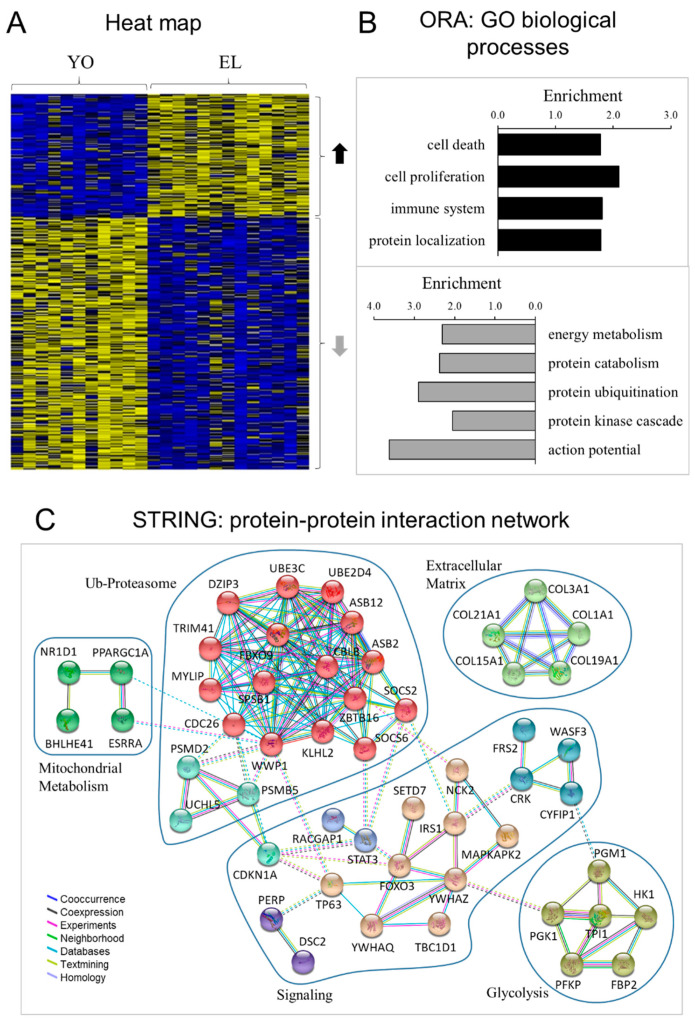
Transcriptomic profiling of healthy aging in men. (**A**) Heat map displaying the relative expression for genes differentially expressed with healthy aging. One hundred and three genes were upregulated, and two hundred and twenty-five were downregulated with age. The heat map depicts the relative expression of each probe set (row) for 11 healthy young (YO) and 13 healthy old (EL) men (columns). The intensity of each block, either yellow (higher expression) or blue (lower expression), represents the magnitude of difference from the mean. (**B**) Over-representation analysis (ORA): top GO biological processes. The lists of genes upregulated (upper) and downregulated (lower) with aging were analyzed for enrichment of specific gene ontology (GO) terms. (**C**) Protein–protein interaction network represented by all genes differentially expressed with aging. The interaction map was generated using STRING with high confidence of 0.7 and all criteria for linkage (co-occurrence, co-expression, experiments, neighborhood, databases, text-mining, and homology). The genes used in this network are listed in Table S1.

Comparison of whole-genome expression profiling between healthy YO and EL muscles indicated that 328 genes were significantly associated with healthy aging alone. Of these, 103 were upregulated and 225 were downregulated with age (Figure 1A), and the full list of differentially expressed genes is given in Table S1. 

To identify the biological processes associated with genes differentially expressed with healthy aging, we performed over-representation analysis (ORA) [47] and independently evaluated up- and downregulated genes using functional annotation tools (adjusted *p* < 0.05; >4 genes) (Figure 1B). Compared with YO, EL had higher transcript levels for genes involved in 21 Gene Ontology (GO) terms, and these gene functional groups reflected four overriding biological processes: cell death and proliferation, immune system and protein localization. Thirty-five GO terms were enriched in genes downregulated with age, and these gene functional groups represented three biological processes: energy metabolism (i.e., glycolysis), protein metabolic process (i.e., protein catabolism and ubiquitination), and signaling (i.e., kinase cascade and action potential). The full list of the significantly regulated GO terms and associated differentially expressed genes are shown in Table S3. Of note, among all signaling pathways implicated in muscle atrophy (Akt/mTOR, BMP-Smad, ATF4-p21, Wnt, Sestrins) [48,49], few mRNAs were differentially expressed with healthy aging, e.g., IRS1 (−1.3 fold), FOXO3 (+1.6), and CDKN1A/p21 (+1.4). The protein–protein interaction network represented by all genes differentially expressed with aging is visualized in Figure 1C. This network revealed interacting clusters for ubiquitin–proteasome, energy metabolism, ECM, and signaling, which emphasized the implication of CDKN1A/p21, STAT3, FOXO3, and IRS1.

Although significant changes in individual genes is critical, minor but concordant changes in sets of functionally related genes is also strongly biologically relevant [47]. Instead of testing differential expression of individual genes (ORA), we also focused on differential expression of pathway-based sets of genes and conducted functional class scoring (FCS) using GeneTrail2 [50]. The FCS comparison of the entire lists of YO versus EL transcripts (Table S4) confirmed ORA analyses, with enriched apoptosis and immune response and depleted protein catabolism in EL muscle. FCS further revealed in EL depleted GO biological processes reflecting protein synthesis and mitochondrial metabolisms and upregulated GO reflecting ECM, fat cell, TNF, and TGFβ signaling. TNF signaling is central for the immune response, and TGFβ signaling impairs muscle regeneration and favors ECM fibrosis. 

In all, these observations are in agreement with our previous histological studies showing that healthy aging is associated with atrophy of type II glycolytic fibers, with a slight accumulation of lipid droplets and with fibrosis of perimysial ECM [17,22].

**Figure 2:**
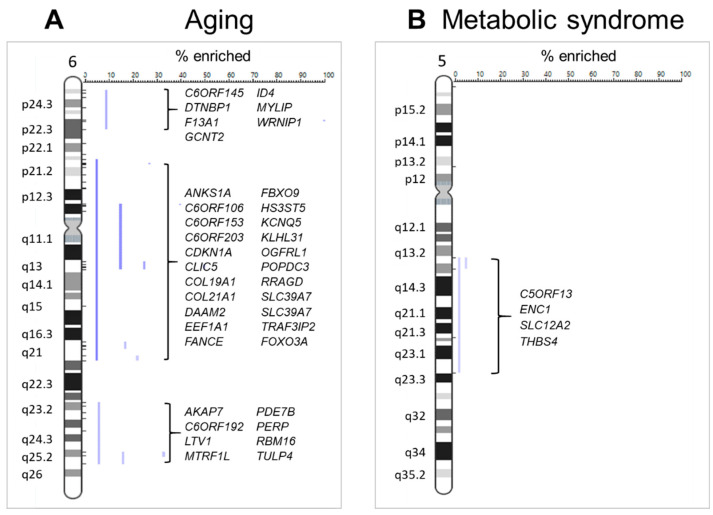
Enriched chromosomal regions. (**A**) Three major regions of chromosome 6 (p24, p21–q21, q23–q25) were significantly enriched in genes differentially expressed with healthy aging. (**B**) One region of chromosome 5 (q13–q23) was significantly enriched in genes differentially expressed with metabolic syndrome in muscle of old men. Blue lines indicate each enriched loci, and the genes associated with the enriched loci are listed for chromosome 5 and 6.

Positional enrichment analysis [51] was then used to delineate chromosomal regions that might be significantly enriched in age-related genes. We found several loci that yielded a significant enrichment score, and the genes associated with these locations can be found in Table S5. Strikingly, 74% of chromosome 6 exhibited significantly enriched regions (p24, p21–q21, q23–q25) (Figure 2A). Of note, chromosome 6q21 was recently described as a longevity-related network hub [52], where FOXO3-centered unit promotes chromatin contacts for co-regulation of neighboring genes. Three of these co-regulated genes were identified in our aging list (ANKS1A, AKAP7, C6Orf145).

### 2.4. Transcriptomic Analysis of Metabolic Syndrome in Old Man Muscle

**Figure 3:**
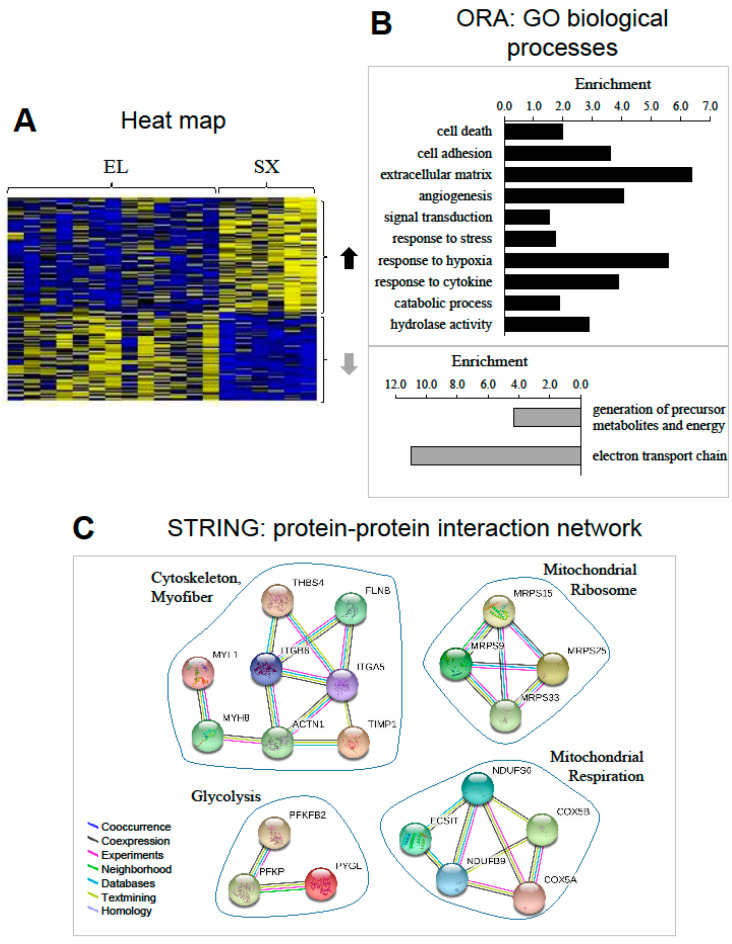
Transcriptomic profiling of metabolic syndrome in old men. (**A**) Heat map displaying the relative expression for genes differentially expressed with metabolic syndrome. Sixty-nine genes were upregulated, and forty-eight were downregulated with metabolic syndrome. The heat map depicts the relative expression of each probe set (row) for 13 healthy old men (EL) and 6 old men with metabolic syndrome (SX) (columns). The intensity of each block, either yellow (higher expression) or blue (lower expression), represents the magnitude of difference from the mean. (**B**) Over-representation analysis (ORA): top GO biological processes. The lists of genes upregulated (upper) and downregulated (lower) with aging were analyzed for enrichment of specific gene ontology (GO) terms. (**C**) Protein–protein interaction network represented by all genes differentially expressed with metabolic syndrome in old men. The interaction map was generated using STRING with high confidence of 0.7 and all criteria for linkage. The genes used in this network are listed in Table S1.

Comparison of whole-genome expression profiling between SX and healthy muscles further indicated that 151 genes were associated with metabolic syndrome (Figure 3A and Table S1). Among them, aging and metabolic syndrome affected gene expression in the same direction for only 26 genes, and even in opposite direction for 8 genes (UCHL1, PSMB5, DISP1, RACGAP1, PFKFB2, PER3, KLF15, MYH8). Moreover, 117 genes were affected only by metabolic syndrome in old men. There was thus a clear distinction between genes differentially regulated by metabolic syndrome and aging.

ORA analysis of the 69 genes over-expressed with metabolic syndrome showed that the dominating biological processes were cell death and adhesion, ECM and angiogenesis, catabolic process and signaling (responses to cytokines and hypoxia). Among the 48 genes under-expressed with metabolic syndrome, the top cluster was related to electron transport chain (Figure 3B). The full list of the significantly regulated GO terms and associated differentially expressed genes is shown in Table S6. The protein–protein interaction network represented by the genes differentially expressed with metabolic syndrome is visualized in Figure 3C. This network similarly revealed interacting clusters for glycolysis, cytoskeleton, and mitochondrial ribosome and respiration.

The FCS comparison of the entire lists of EL versus SX transcripts mostly confirmed analyses of differentially expressed genes (Table S4) and further revealed in SX upregulated GO biological processes reflecting fat metabolism and TGFβ signaling, and depleted GO reflecting translation and protein targeting to the endoplasmic reticulum. Of note, FCS indicated that amino acid metabolism and protein catabolism were downregulated with healthy aging but upregulated with metabolic syndrome in old men.

Overall, these observations are in agreement with our previous histological data showing that metabolic syndrome in the elderly is associated with a strong accumulation of intra-myofibrillar lipid droplets, with fibrosis of endomysial ECM and with an altered capillarization [17,22].

Finally, we examined using positional enrichment analysis whether the SX-related genes were over-represented at genomic loci [51]. Several chromosomes had significant hits (Table S7), and 30% of chromosome 5 (q13–q23) exhibited significantly enriched regions (Figure 2B).

### 2.5. Proteomic Profiling of Chronological Aging and Metabolic Syndrome in Man Muscle

The differential expression profiles of mRNA constitute an essential level of information, but mRNAs only give indications about potential regulations of biological processes, as all mRNAs are not obligatorily translated and resulting proteins can be degraded. Further studies were then performed to investigate the proteomes of YO, EL, and SX muscles.

**Figure 4:**
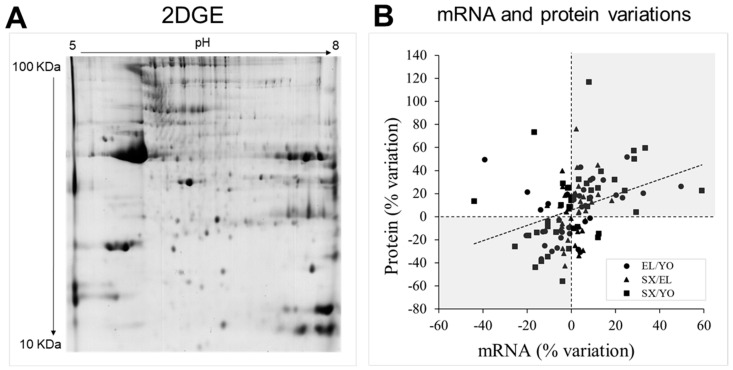
Human muscle proteome. (**A**) Representative 2DGE image obtained from total protein extracts of human vastus lateralis skeletal muscle. 2DGE was performed using a pH range of 5-8. Protein loading was 700 µg, and gels were stained using colloidal Coomassie blue G-250. Differentially expressed and identified proteins are indicated in Figure S3. (**B**) Comparison of mRNA and protein variations between healthy young men (YO), healthy old men (EL), or old men with metabolic syndrome (SX).

In order to evaluate changes in muscle proteome associated with age and/or metabolic syndrome, total protein extracts from 15 YO, 15 EL, and 9 SX biopsies were resolved by 2DGE (Figure 4A and Figure S3). Each extract was assessed separately in duplicate and seventy-eight gels with medium range IPGs (immobilized pH gradient strips) (pH 5–8) were analyzed. 2DGE revealed 586 protein spots that were matched between all individuals. Seventy-five protein spots were found to be differentially expressed and sixty-three were identified by mass spectrometry (Table 2), corresponding to thirty-nine different proteins. Aging was associated with the differential expression of 40 different spots, corresponding to 24 different proteins (13 increased and 11 decreased with aging). Metabolic syndrome in old men was associated with the differential expression of 28 different spots, corresponding to 24 different proteins (13 increased and 11 decreased with metabolic syndrome). Among them, five spots changed with both aging and metabolic syndrome; they corresponded to five proteins, of which three varied in an opposite manner with aging and metabolic syndrome. 

For each YO, EL, and SX subject, the same muscle biopsy was used for both transcriptomic and proteomic analyses, and we thus compared the levels of proteins and corresponding mRNAs between subjects. Taking into account each particular protein–mRNA couple independent of YO, EL, and SX groups, 33% of proteins were (and 15% tended to be) correlated with their corresponding mRNA level (Figure S4). Most correlations were positive, and one notable exception was the negative correlation between protein and mRNA levels for Fructose-1,6-bisphosphatase isozyme 2 (FBP2).

When we compared changes in mRNA and protein levels between YO, EL, and SX groups (Figure 4B), we observed a significant but weak correlation (*r* = 0.34, *p* < 0.001) between variations in protein and mRNA levels. However, despite weak correlation, 66% of mRNA and protein changed in the same direction between groups (grey in Figure 4B). 

These observations emphasize the importance of studying the proteome. Because cellular proteins constitute the true effectors of biological processes, a detailed analysis of the expression profiles of proteins was then performed, and Table 2 summarizes the main properties of the proteins differentially regulated in skeletal muscle between YO, EL, and SX men.

### 2.6. Perturbations of the Myofilaments Networks during Aging with or without Metabolic Syndrome

Skeletal muscle contraction depends on interactions between myosin-thick and actin-thin filaments. In our study, muscle aging was associated with higher levels of two isoforms of a myosin light-chain (MYL6B), which is prominently expressed in slow-twitch muscles, while SX muscle exhibited higher level of MYL2. In addition to myosin, the thick filament contains important regulatory proteins, such as MYBPC1, which was selectively downregulated in SX compared with the EL muscles. MYBPC1 has significant effects on length, thickness, and lateral alignment of myosin filaments [53]. 

With regard to the thin filament, aging was associated with the downregulation of two isoforms of fast troponin T (TNNT3) and, conversely, with the upregulation of four isoforms of slow troponin T (TNNT1). In contrast to aging, metabolic syndrome was associated with an increased expression of two isoforms of fast skeletal troponin C (TNNC2). TNNC is the Ca2+-binding subunit of the troponin complex, and its interaction with troponins I and T is central to the regulation of contraction [54]. In addition to the troponin complex, the thin filament contains other proteins that anchor actin filaments to the Z-disk and regulate its growth [55]. In SX muscles, expressions of two actin-binding proteins, CAPZA2 and CFL2, were increased. By capping the barbed end of actin filaments, CAPZA2 regulates the growth of the actin filament [56]. CFL2, a member of the actin depolymerization factors [57], is also critical in the regulation of actin filament dynamics.

**Figure 5:**
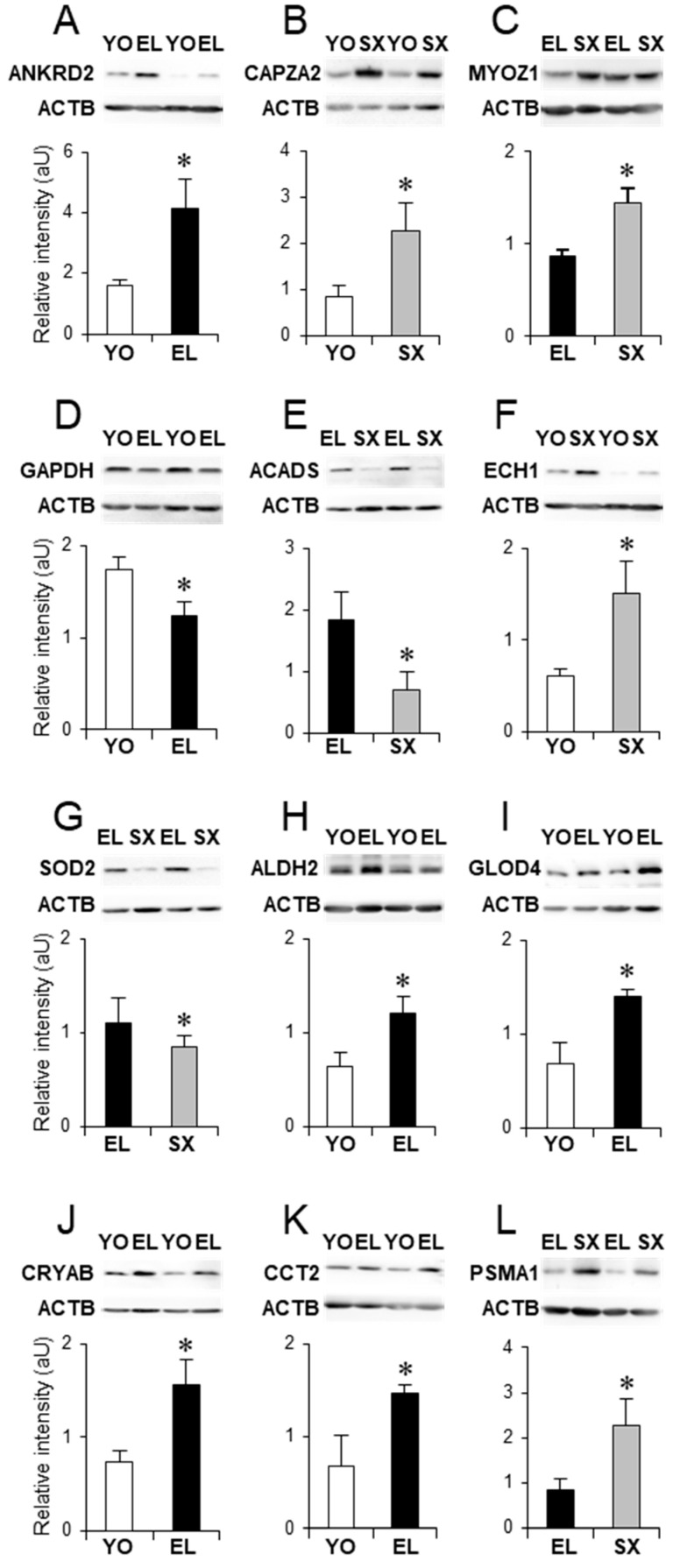
Proteins differentially expressed with healthy aging and metabolic syndrome in old men. Examples of differential expression of proteins associated with myofibrillar filaments (**A**–**C**), energy metabolism (**D**–**F**), cytoprotection and cytodetoxification (**G**–**K**), and proteolysis (**L**). In each panel, a representative Western blot is shown, and the histogram represents Western blot quantification (*n* = 7) for young (YO), healthy old men (EL), and old men with metabolic syndrome (SX). The protein names are listed in Table 2. Results are shown as means ± SE. * *p* < 0.05 indicates significant difference between groups.

Myofibrillar-associated proteins can also participate in signal transduction cascades. Aging was associated with an increased expression of ANKRD2. ANKRD2 is preferentially expressed in slow type I fibers and is a mechano-sensing protein that links myofibrillar stress response to muscle gene expression [58]. Western-blotting (WB) experiments confirmed the overexpression of ANKRD2 and CAPZA2 in skeletal muscle of EL and SX men, respectively (Figure 5A,B).

The slow- and fast-twitch fiber composition of skeletal muscle is tightly regulated, and the phosphatase calcineurin plays a critical role in this process [59]. In the present study, we found that aging was associated with a downregulation of myozenin-1 (MYOZ1, or calsarcin-2), which is expressed in fast-twitch fibers and is central for calcineurin regulation [59]. In contrast, metabolic syndrome was associated with an increased expression of MYOZ1 compared with healthy elderly, which was confirmed by WB (Figure 5C).

In all, the present findings report several modifications in sarcomeric actomyosin and regulatory proteins, which could contribute to our previously observed fiber deformation [17] and to alterations of contractile properties in the old muscle. An increased expression of MYL6B, TNNT1, and ANKRD2, together with the downregulations of TNNT3 and MYOZ1, is consistent with a fast-to-slow transition of the old skeletal muscle [60]. Conversely, the upregulations of TNNC2 and MYOZ1, and downregulation of MYBPC1, suggest that such transition is more limited in aged muscle with metabolic syndrome.

### 2.7. Perturbations in Energy Metabolism during Aging with or without Metabolic Syndrome

Our proteomic analysis indicates that aging and metabolic syndrome in men are associated with disturbance in muscle energy metabolism, including phosphocreatine shuttle, anaerobic glycolysis, NADH/NAD^+^ shuttle, and lipid metabolism.

#### 2.7.1. Anaerobic Glycolysis

Several enzymes involved in anaerobic metabolism were downregulated with healthy aging. Among these, five isoforms of muscle PYGM decreased in EL compared with YO muscle. PYGM catalyzes the phospholytic cleavage of glycogen to produce glucose-6-phosphate, the major substrate of glycolysis. Four isoforms of TPI1 that catalyze the isomerization of the dihydroxyacetone phosphate (DHAP) and D-glyceraldehyde-3-phosphate were downregulated with aging. D-glyceraldehyde-3-phosphate was then converted to 1,3-diphosphoglycerate by GAPDH, which decreased in EL muscle, as also shown by WB (Figure 5D). Enolase catalyzes the conversion of 2-phosphoglycerate to phosphoenolpyruvate. In old men, 3 isoforms of ENO3 were downregulated with aging. Finally, we also detected lower levels of PKM, which catalyzes the last step in glycolysis and is responsible for net ATP production. In the elderly, metabolic syndrome was also associated with a downregulation of several glycolytic enzymes. Indeed, isoforms of PYGM, TPI1, ENO1, ENO3, and PKM decreased in skeletal muscle of old men with metabolic syndrome.

Overall, these proteomic observations confirm our transcriptomic data and suggest that aging and metabolic syndrome in men are associated with a downregulation of glycolysis. FBP2 catalyzes the reverse of the phosphofructokinase (PFK) reaction early in glycolysis. High activity of one of these two enzymes (FBP2 or PFK) is accompanied by low activity of the other, and this process is central for the regulation of glycolysis. In agreement with a reduced glycolysis, FBP2 increased with aging but was normalized in SX muscle, which suggests that the downregulation of glycolysis is stronger in healthy EL than in SX muscle.

#### 2.7.2. NADH/NAD^+^ Shuttle

NADH formed by glycolysis not only participates in reduction–oxidation reactions as co-substrates but also plays important roles in metabolic regulation in both cytosol and mitochondria [61]. The effective transport of NADH/NAD^+^ between cytosol and mitochondria is accomplished by shuttle systems. Our results show that metabolic syndrome in the elderly was associated with a decreased expression of GOT1. This enzyme catalyzes the interconversion of aspartate and α-ketoglutarate to oxaloacetate and glutamate in the cytosol [62], and GOT1 downregulation suggests that NADH/NAD^+^ shuttle is less efficient in the old SX muscle.

#### 2.7.3. Lipid Metabolism

In addition to carbohydrates, lipids are another important source of energy for the skeletal muscle. Fatty acid binding proteins (FABPs) enhance the transport of fatty acids from the cell membrane to the sites of oxidation and esterification [63]. Metabolic syndrome in elderly men was associated with the upregulation of FABP3, the major FABP in skeletal muscle [64]. Metabolic syndrome was also associated with a downregulation of mitochondrial ACADS, which catalyzes the initial step in each cycle of fatty acid β-oxidation [65]. The degradation of unsaturated fatty acids by β-oxidation requires several auxiliary enzymes, such as ECH1. One ECH1 isoform was upregulated in SX muscle, while another ECH1 isoform also increased in EL muscle. WB confirmed the differential expression of ACADS and ECH1 in SX compared with EL and YO muscles, respectively (Figure 5E,F).

These results suggest that aging and particularly metabolic syndrome were associated with alterations in mitochondrial β-oxidation, and they provide some mechanistic clues to the accumulation of intramyocellular lipid droplets that we previously observed by histochemistry [17].

### 2.8. Cytoprotection and Cytodetoxification in Old Muscle with or without Metabolic Syndrome

Reactive oxygen species (ROS) are byproducts of normal cellular metabolism, and ROS can cause cellular damage by oxidation of lipids, proteins, and nucleic acids [5]. Several enzymes, including superoxide dismutases (SOD) and peroxiredoxins (PRDX), are involved in reducing the oxidative damage. Our proteomic data indicate that aging was associated with the downregulation of PRDX2. Moreover, mitochondrial SOD2 was downregulated in SX muscle, which was confirmed by WB (Figure 5G) and could contribute to insulin resistance in such individuals [66].

Increased ROS may lead to production of cytotoxic aldehydes, and several families of detoxification enzymes, including aldehyde dehydrogenase (ALDH), provide protection against reactive aldehydes. Our results indicate that mitochondrial ALDH2 was upregulated with aging, which was confirmed by WB (Figure 5H). In contrast, SX muscle exhibited a decreased expression of another aldehyde dehydrogenase (ALDH9A1), suggesting increased reactive aldehydes in these individuals. Toxic aldehydes can also be produced by glycolysis. TPI1 ensures that DHAP produced by aldolase is metabolized by glycolytic enzymes, and impairment in TPI1 may result in conversion of DHAP into methylglyoxal, a toxic aldehyde eliminated by the glyoxalase system [67]. Accordingly, glyoxalase domain-containing protein 4 (GLOD4) was more abundant in EL muscle, and this was confirmed by WB (Figure 5I).

In all, these observations suggest an increased ROS production in old (EL and SX) compared with YO muscles, but a better detoxification of cytotoxic aldehydes in EL than in SX muscles.

### 2.9. Proteostasis in Old Muscle with or without SX

Against cellular stress, quality control of protein folding and homeostasis represents a fundamental cellular activity. Our proteomic analysis demonstrated an upregulation of several small heat shocks proteins (HSPB): HSPB6 (or HSP20) in EL muscle and HSPB1 (or HSP27) and CRYAB (or HSPB5) in SX muscle. These HSPBs are ATP-independent chaperones that prevent the aggregation of improperly folded proteins, or that are involved in their transfer to proteolytic systems (proteasomes or lysosomes). In addition to HSPB proteins, the chaperonin CCT2 also increased with aging; CCT2 assists the folding of newly translated polypeptides through multiple rounds of ATP-driven release and rebinding of partially folded intermediate forms. Substrates of HSPB1, HSPB5, HSPB6, and CCT2 include cytoskeletal proteins [68]. WB confirmed the differential expressions of CRYAB and CCT2 between YO and EL muscles (Figure 5J,K). Therefore, despite increased ROS production, upregulation of these chaperones may contribute to maintain cytoskeletal and myofibrillar integrity during aging.

The ubiquitin-proteasome system (UPS) is a major non-lysosomal proteolytic system [69]. Ubiquitin carboxyl-terminal hydrolase 14 (USP14) is required for recycling ubiquitin to maintain proteolysis by the proteasome [70], and our analysis identified a decreased expression of USP14 during aging. In contrast, we also report the upregulation of PSMA1 and PSMB4, two subunits of the 20S core proteasome in SX muscle. WB confirmed the differential expression of PSMA1 between EL and SX muscles (Figure 5L).

These results are in agreement with our transcriptomic analysis and may suggest that proteolysis is downregulated with chronological aging but upregulated with metabolic syndrome in old subjects.

### 2.10. Perturbations in Membrane Repair in the Old Skeletal Muscle

Repair of damage to the plasma membrane is critical for cellular physiology, and distinct contributors were described in the literature [71]. Annexin A5 (ANXA5) self-assembles into two-dimensional arrays on membranes and may contribute to membrane repair [72]. ANXA5, which decreased with aging, also participates in numerous membrane-related processes, including exo-and endocytosis and vesicle trafficking [71].

Another process involves translocation of intracellular vesicles to the injury site to form a membrane repair patch composed of several proteins. In skeletal muscle, TRIM72 (also called MG53) is an essential component of this membrane repair patch [73], and PTRF anchors TRIM72 to the injury site [74]. Our present findings report an upregulation of TRIM72 and PTRF with aging, suggesting that expression of these proteins could protect against damage to the plasma membrane in skeletal muscle during aging. To our knowledge, no previous study of aged muscle has reported the differential expression of TRIM72 and PTRF.

## 3. Materials and Methods

### 3.1. Subjects Characteristics

Our study included 15 healthy young adult (YO, 21 y) men, 15 healthy elderly (EL, 73 y) men, and 9 elderly men diagnosed with metabolic syndrome (SX, 73 y) for at least 10 years. YO and EL subjects were all in good health, as evidenced by physical and clinical examinations. EL and SX men were selected from the PROOF cohort [40,75]. Exclusion criteria were prior myocardial infarction or stroke, heart failure, atrial fibrillation, type 2 diabetes, morbid obesity (body mass index > 35 kg/m^2^), Parkinson’s disease, and any other disease limiting life expectancy to less than 5 years. Dependent elderlies or those living in an institution were excluded. YO men were healthy volunteers with the same exclusion criteria as the elderlies. All subjects underwent standard medical examination and performed a maximal stress exercise before their inclusion in the study. Metabolic syndrome was defined according to [76] and was diagnosed when 3 of 5 components occurred: waist circumference ≥ 102 cm, triglycerides ≥ 1.7 mmol/L, HDL cholesterol <1.04 mmol/L, blood pressure ≥ 130/85 mm Hg, and/or fasting glucose ≥ 5.6 mmol/L. Daily energy expenditure was assessed according to [77]. Body composition was measured using dual X-ray absorptiometry (DEXA, Hologic QDR-2000). VO_2peak_ was estimated using a cycle ergonometer according to [77], and maximal knee extension isometric and specific strength were measured using a Cybex II (Ronkonkoma) as described in [78]. All measurements were taken from the subject’s right leg, subsequently biopsied.

### 3.2. Sample Preparation

All subjects received standard meals on the day before the study, and biopsies were collected between 9 and 10 a.m., after an overnight fast. For each subject, a single needle biopsy was taken under local anesthesia from the superficial portion of the left vastus lateralis using a percutaneous technique [79]. Each biopsy was divided into 3 samples and either (i) pretreated with RNAlater (Ambion) overnight at 4 °C and stored at −80 °C for transcriptomics, (ii) snap-frozen in liquid N_2_ for proteomics, or (iii) frozen in isopentane cooled on liquid N_2_ and stored at −80 °C for histology [17,22].

### 3.3. Microarray Hybridization and Transcriptomic Analysis

Independent RNA isolations were carried out for each biopsy, but a sufficient amount of high quality RNA could not be obtained for 4 YO, 2 EL and 3 SX samples. Independent hybridization reactions were then performed to generate data sets for 11 YO, 13 EL and 6 SX muscles. TissueRuptor and RNeasy Fibrous Tissue Mini Kit (Qiagen) were used to isolate muscle RNA. Integrity of RNA samples was verified using an Agilent Bioanalyser with the RNA 6000 Nano labchip^®^ kit (Agilent). Concentration and purity were determined with a Nanodrop ND-1000 spectrophotometer. The depletion of the ribosomal fractions was processed using 2 µg of total RNA and magnetic beads from Ribominus Kit (Invitrogen). Biotinylated single stranded cDNA were prepared from 400 ng of depleted total RNA according to the Affymetrix WT protocol. Following fragmentation and terminal labeling, 5.5 µg of single stranded cDNA was hybridized for 16 h at 45 °C and 60 rpm on GeneChip^®^ Human Exon 1.0 ST Array in the Affymetrix Oven 645. GeneChips were washed and stained in the Affymetrix Fluidics Station 450 with Hybridization Wash and Stain Affymetrix kit. GeneChips were scanned using the Affymetrix GeneChip Scanner 3000 7G, and data were generated with Affymetrix Expression Console v 1.2.1 software using RMA algorithm. Data were processed using GeneSpring GX 13 (Agilent) and analyzed by one-way ANOVA followed by pairwise comparisons using Tukey HSD post-hoc test. Benjamini–Hochberg multiple testing correction was performed to identify differentially expressed entities with a significance threshold set at *p* < 0.05. The data discussed in this publication have been deposited in NCBI’s Gene Expression Omnibus [80] and are accessible through GEO Series accession number GSE136344 (https://www.ncbi.nlm.nih.gov/geo/query/acc.cgi?acc = GSE136344; release date 14 July 2021).

Biological replicates showed Pearson’s correlation coefficients above 0.95, indicating high reproducibility of the gene expression data (Supplementary Materials Figure S1). To confirm microarray analyses, quantitative real time PCR (qRT-PCR) was carried out for EEF1A1, TSC22D1, KCNMA1, MYOZ2, LRP1B, SLC43A2, PDLIM1, PER3, MYLIP, KLF15, GPCPD1, and IGFN1 mRNAs and 18S RNA as housekeeper. Reverse transcription of total RNA was performed using the QuantiTect Reverse Transcription kit (Qiagen) and qPCR using the FastStart DNA Master SYBR Green I kit (Roche), a CFX96 thermocycler (Biorad), and the comparative CT method. qRT-PCR validated microarray analyses and a Pearson’s correlation of 0.96 (*p* < 4.5 × 10^−13^) was observed between the fold changes from the microarray and the qRT-PCR analyses (Figure S2). 

We tested the significance of the overlap between our set of differentially expressed genes with other databases using a hypergeometric test (http://nemates.org/MA/progs/overlap_stats.html, access on 15 April 2021) and a background of 18,738 genes. For over-representation analysis (ORA), the lists of genes up- and downregulated with aging or metabolic syndrome were analyzed for enrichment of specific Gene Ontology (GO) terms using Genomatix software suite v3.2 and adjusted for multiple testing correction according to [81]. The protein–protein interaction network represented by differentially expressed genes was investigated using the Search Tool for Retrieval of Interacting Genes (STRING, version 11.0) database (https://string-db.org, access on 15 April 2021) [82]. STRING analysis options were based on ‘evidence’ mode, high confidence (0.7), and we used MCL clustering and 1.8 inflation to reveal sub-grouping within the networks. Functional class scoring of pathway-based sets of genes (FCS) was performed using GeneTrail2 web service [50] and a Kolmogorov–Smirnov test with Benjamini–Yekutieli correction (*p* < 0.01) for multiple testing.

### 3.4. Two-Dimensional Gel Electrophoresis (2DGE)

Total muscle extracts were prepared for each subject, and each individual (15 YO, 15 EL, and 9 SX) was assessed separately. Muscle aliquots were homogenized (40 mg/mL) in a solubilization buffer containing 8.3 M urea, 2 M thiourea, 2% (*w*/*v*) CHAPS, 1% (*v*/*v*) dithiothreitol, and 2% (*v*/*v*) IPG buffer pH 3–10 using a TissueRuptor (Qiagen), shaken for 30 min on ice and centrifuged for 30 min at 10,000× *g*. The supernatants were aliquoted and stored at −20 °C until analysis. Protein concentration, determined after a 500-fold dilution and using the Bradford assay system (Bio-Rad), was 6.5 ± 1.2 mg/mL, 6.7 ± 0.8 mg/mL, and 6.7 ± 0.9 mg/mL for YO, EL, and SX extracts, respectively.

For each individual, 700 µg protein was separated using 18 cm ReadyStrip IPG strips (pH 5–8) and Electrode Wicks (Bio-Rad). For isoelectrofocusing, samples were diluted with rehydration buffer containing 8.3 M urea, 1 M thiourea, 2% (*w*/*v*) CHAPS, 0.28% (*v*/*v*) dithiothreitol, 2% (*v*/*v*) IPG buffer (pH 3–10), and 0.01% (*w*/*v*) Coomassie Brilliant blue R-250. The IPG strips were passively rehydrated with 330 µl of this solution at room temperature for 16 h under mineral oil in the PROTEAN IEF Cell system (Bio-Rad) at 20 °C and actively rehydrated using Electrode Wicks loaded onto IPG strips for 6 h at 50 V. During active rehydration, Electrode Wicks were changed every 2 h. Isolectrofocusing was then performed at 0.05 mA per IPG strip at 50 V for 2 h, 200 V for 1 h, 500 V for 1 h, 1000 V for 2 h, 8000 V for 6 h, and finally 8000 V to achieve 46,000 Vh.

The strips were then equilibrated twice for 15 min with gentle shaking in equilibration buffer containing 6 M urea, 50 mM Tris-HCl buffer (pH 8.8), 30% (*v*/*v*) glycerol, 2% (*w*/*v*) sodium dodecyl sulfate. Dithiothreitol (1% *w*/*v*) was added to the first, and iodoacetamide (5% *w*/*v*) was added to the second equilibration buffer. Separation according to protein mass was carried out using a Protean Plus DodecaCell system (Bio-Rad) on homogenous 20 cm polyacrylamide gels containing 11% (*w*/*v*) total acrylamide and a 2.6% (*w*/*w*) ratio of bis to total acrylamide. The equilibrated strips were sealed to the top of the horizontal gel with agarose and subjected to 50 V for 1 h followed by 9 mA per gel until the blue dye reached the bottom of the gel. 

2DGE gels were fixed overnight in a solution containing 30% (*v*/*v*) ethanol and 2% (*v*/*v*) orthophosphoric acid, washed twice for 30 min in 2% (*v*/*v*) orthophosphoric acid, and then transferred to a solution containing 18% (*v*/*v*) ethanol, 2% (*v*/*v*) orthophosphoric acid, and 15% (*v*/*v*) ammonium sulphate for 30 min. The gels were stained for 72 h with 0.06% (*w*/*v*) Coomassie Blue G-250 added to this last solution. Gels were scanned using the ImageScanner and LabScan-v.5 software (Amersham), and protein spots were analyzed and matched between all gels (duplicates and conditions) using Progenesis SameSpot software (Non Linear Dynamics). Proteins with significant changed abundance were picked for tryptic digestion from gels, and a single spot from the most representative gel was used for each identification.

### 3.5. Protein Identification by Mass Spectrometry

Excised protein spots from 2DGE gels were destained with 25 mM ammonium bicarbonate, 5% (*v*/*v*) acetonitrile for 30 min and twice in 25 mM ammonium bicarbonate, 50% (*v/v*) acetonitrile for 30 min each. Protein spots were then dehydrated using 100% acetonitrile for 10min and were completely dried using a Speed Vac. Proteins were digested overnight at 37 °C using 10 ng/µL of sequence grade-modified trypsin (Promega) in 25 mM ammonium bicarbonate. Peptide extraction was optimized by adding 100% acetonitrile, followed by 15 min of sonication.

For liquid chromatography coupled with tandem mass spectrometry (LC-MS/MS), peptides mixtures were analyzed by on-line nanoflow liquid chromatography using the Ultimate 3000 RSLC (Dionex) with 15 cm nanocapillary columns of an internal diameter of 75 µm (Acclaim Pep Map RSLC, Dionex, Sunnyvale, CA, USA). The gradient consisted of 4–50% (*v*/*v*) acetonitrile in 0.5% (*v/v*) formic acid at a flow rate of 300 nL/min for 30 min. The eluate was electrosprayed into an LTQ Velos (Thermo Fisher Scientific, Waltham, MA, USA) through a nanoelectrospray ion source. The LTQ Velos was operated in a CID top 10 mode. Raw files were processed using version 2.4 of Thermo Proteome Discoverer. For protein identification, the NCBInr suscrofa protein database (41,000 seq) was combined with the sequences of human keratin contaminants. Peptide mass tolerance was set to 1.5 Da, and fragment mass tolerance was set to 0.8 Da. Two missed cleavages were allowed. Variable modifications were methionine oxidation (M) and carbamidomethylation (C) of cysteine. Protein identification was validated when at least three peptides originating from one protein showed significant identification Mascot scores (*p* < 0.05). In the present study we considered that proteins validated for the whole analyzed spots were really present on the basis of score, peptide number related to each protein, and MW (molecular weight) agreements.

### 3.6. Immunoblotting

For Western blot (WB) analysis, total protein extracts were resolved by sodium dodecyl sulfate polyacrylamide gel electrophoresis and electrotransferred to Hybon^TM^-P membrane and probed with antibodies against CAPZ2, ANKRD2, CCT2, MYOZ1, GAPDH, ECH1, ACADS, ALDH2, CRYAB, SOD2, GLOD4, and PSMA1 (all from Genetex, Irwin, CA, USA). Primary antibodies were detected with horseradish peroxidase (HRP)-linked goat anti-mouse or anti-rabbit secondary antibodies (Santa Cruz, Dallas, TX, USA) using enhanced chemiluminescence with LuminataTM Western HRP Substrate (Millipore) and a charge coupled device camera (GBOX, Syngene, CA, USA). Each blot was dehybridized using 1X ReBlot Plus Strong antibody stripping solution (Millipore, Sigma-Aldrich, St. Louis, MO, USA) and probed with anti-ACTB (β-actin, Santa Cruz) for normalization.

### 3.7. Other Statistical Analyses

Unless otherwise stated, values are means ± SE. YO, EL, and SX groups were compared using one-factor analysis of variance, followed by post hoc Tukey’s test for pairwise comparisons between groups. Statistical analyses were performed using XLSTAT (Addinsoft, Saugus, MA, USA) and significance was set at *p* < 0.05.

## 4. Conclusions

Our previous analysis of muscle histology [17,22], combined with the present investigation of the muscle transcriptome and proteome, provide an integrated view of muscle healthy aging in men and further identify some specific alterations associated with metabolic syndrome. To our knowledge, this is the first coordinated proteomic and transcriptomic analysis investigating muscle changes associated with aging and/or metabolic syndrome in old men.

Whole genome expression profiling of mRNAs indicate that aging and metabolic syndrome were both associated with increased cell death, activation of the immune response, ECM fibrosis, and altered lipid metabolism. Both aging and metabolic syndrome revealed a decline in energy metabolism; however, this decline essentially affected glycolysis for healthy aging and mitochondrial metabolism for aging with metabolic syndrome. Our transcriptomic analysis also pointed to a striking inversed regulation of amino acid metabolism and protein catabolism that both declined with aging but raised with metabolic syndrome.

In contrast to whole-genome transcriptomics, proteomics provides only a restricted view of the proteome. Nonetheless, we identified some correlations between changes in transcriptome and proteome levels, and our proteomic observations globally confirmed our transcriptomic data. The proteome of muscle aging in men revealed several modifications in sarcomeric proteins, which may contribute to alterations in contractile properties. Proteome data are also consistent with a fast-to-slow transition and a downregulation of glycolysis in the old skeletal muscle; however, these transitions appear stronger in healthy EL than in SX muscle. Our proteomic observations further indicate an age-related increase in ROS production, but nonetheless a better detoxification and membrane repair in EL than in SX muscles.

These results are in agreement with our immunohistochemical analyses. They indicate potential mechanisms of aging and could lead to development of biomarkers that may be targets for the comprehension, prevention, and treatment of sarcopenia and metabolic syndrome.

## Figures and Tables

**Table 1:** Subject Characteristics.

		YO (*n* = 15)	EL (*n* = 15	SX (*n* = 9)
Age (y)		21.8 ± 0.9	73.5 ± 0.2 *	72.9 ± 0.8 *
Body weight (kg)		74.0 ± 2.3	69.5 ± 2.3	92.5 ± 4.0 *^,†^
BMI (kg/m^2^)		22.8 ± 0.8	24.6 ± 0.8	30.7 ± 0.4 *^,†^
VO_2peak_ (mL/min/kg)		38.3 ± 1.6	25.7 ± 1.3 *	13.3 ± 1.3 *^,†^
Blood pressure ^§^ (mm Hg)	systolic	118.1 ± 2.4	134.2 ± 3.3 *	156.3 ± 7.7 *^,†^
	diastolic	75.7 ± 2.0	82.8 ± 2.0 *	100.0 ± 2.8 *^,†^
Specific strength (N/m^3^)		11.1 ± 0.4	7.9 ± 0.2 *	7.8 ± 0.5 *
Daily energy expenditure (kJ/kg/day)			147 ± 4	139 ± 7
Waist circumference ^§^ (cm)			86.8 ± 2.3	104.2 ± 2.9 ^†^
Glucose ^§^ (mmol/L)			4.97 ± 0.09	8.88 ± 1.65 ^†^
Lipid levels (mmol/L)	HDL cholesterol ^§^		1.51 ± 0.10	1.32 ± 0.16
	Triglycerides ^§^		0.98 ± 0.08	1.82 ± 0.40 ^†^
Body fat mass (% body weight)			21.6 ± 1.3	31.7 ± 1.6 ^†^
Appendicular lean mass (% body weight)			31.9 ± 0.7	27.0 ± 0.6 ^†^

Data are presented as mean + SE for young (YO), elderly (EL) and old men with metabolic syndrome (SX). BMI, body mass index; HDL, high-density lipoprotein; * *p* < 0.05 vs. YO; ^†^ *p* < 0.05 vs. EL; ^§^ metabolic syndrome criteria.

**Table 2:** Identification of differentially expressed protein in vastus lateralis muscle of young (YO), healthy elderly (EL) and elderly with metabolic syndrome (S).

Spot n°	Accession	Symbol	Protein	Anova (P)	Fold Change			Score	% Cov	PSM	Unique Peptides
					EL vs. YO	EL vs. SX	YO vs. SX				
**Myofilaments and cytoskeleton**											
1820	P14649	MYL6B	Myosin light chain 6B	<0.001	1.48		1.49	708	46	26	12
1851	P14649	MYL6B	Myosin light chain 6B	<0.001	1.57		1.92	544	51	39	12
979	P10916	MYL2	Myosin regulatory light chain 2, ventricular/cardiac muscle isoform	0.003		1.76	2.17	928	74	32	10
1930	Q00872	MYBPC1	Myosin-binding protein C, slow-type	0.019		−1.25		2236	36	90	32
714	P68133	ACTA1	Actin, alpha skeletal muscle	0.003	−1.24	1.19		395	22	12	6
683	P68133	ACTA1	Actin, alpha skeletal muscle	0.001		1.29		274	17	9	5
754	P45378	TNNT3	Troponin T, fast skeletal muscle	0.004	−1.20		−1.34	808	33	28	9
1926	P45378	TNNT3	Troponin T, fast skeletal muscle	0.015	−1.45			1219	36	82	11
1825	P13805	TNNT1	Troponin T, slow skeletal muscle	<0.001	1.52		1.43	1923	32	60	8
1826	P13805	TNNT1	Troponin T, slow skeletal muscle	<0.001	1.76			349	32	14	8
1859	P13805	TNNT1	Troponin T, slow skeletal muscle	0.005	1.17		1.18	1756	32	92	11
1873	P13805	TNNT1	Troponin T, slow skeletal muscle	0.026	1.15		1.19	1501	32	65	10
962	P02585	TNNC2	Troponin C, skeletal muscle	0.003		1.43	1.74	139	26	5	3
793	P47755	CAPZA2	F-actin-capping protein subunit alpha-2	0.013			1.29	744	38	22	7
988	Q9Y281	CFL2	Cofilin-2	0.002		1.20	1.16	1197	47	32	7
745	Q9GZV1	ANKRD2	Ankyrin repeat domain-containing protein 2	0.042	1.26			884	39	29	12
1844	Q9NP98	MYOZ1	Myozenin-1	0.002	−1.28	1.40		276	25	6	4
**Energy metabolism**											
1941	P06732	CKM	Creatine kinase M-type	0.006	1.17		1.25	2823	50	132	17
330	P11217	PYGM	Glycogen phosphorylase, muscle form	0.024	−1.37			2683	50	116	35
341	P11217	PYGM	Glycogen phosphorylase, muscle form	0.001	−1.42		−1.55	1418	32	47	24
350	P11217	PYGM	Glycogen phosphorylase, muscle form	0.009	−1.28		−1.28	1885	39	62	28
353	P11217	PYGM	Glycogen phosphorylase, muscle form	<0.001	−1.27		−1.34	3085	47	114	36
355	P11217	PYGM	Glycogen phosphorylase, muscle form	0.011	−1.39		−1.52	1329	37	47	26
340	P11217	PYGM	Glycogen phosphorylase, muscle form	0.021			−1.81	1087	24	36	17
347	P11217	PYGM	Glycogen phosphorylase, muscle form	0.015			−1.58	515	11	15	8
1842	P60174	TPI1	Triosephosphate isomerase	<0.001	−1.30		−1.47	1063	49	30	11
1862	P60174	TPI1	Triosephosphate isomerase	<0.001	−1.18		−1.15	1543	66	63	13
1879	P60174	TPI1	Triosephosphate isomerase	0.009	−1.23		−1.33	3833	77	177	18
1845	P60174	TPI1	Triosephosphate isomerase	<0.001	−1.17	−1.30	−1.51	1827	66	83	14
1841	P60174	TPI1	Triosephosphate isomerase	0.001		−1.22	−1.36	2676	58	76	14
773	P04406	GAPDH	Glyceraldehyde-3-phosphate dehydrogenase	0.007	−1.18			1563	42	42	11
748	P13929	ENO3	Beta-enolase	<0.001	−1.24		−1.23	2358	26	65	9
1857	P13929	ENO3	Beta-enolase	0.007	−1.40			1604	38	57	12
1874	P13929	ENO3	Beta-enolase	0.006	−1.24		−1.27	2395	52	85	17
672	P13929	ENO3	Beta-enolase	0.003		−1.28	−1.37	1356	39	37	13
677	P13929	ENO3	Beta-enolase	0.002			−1.35	1863	40	53	13
658	P06733	ENO1	Alpha-enolase	0.008			−1.27	1351	46	38	14
561	P14618	PKM	Pyruvate kinase isozymes M1/M2	0.001	−1.39		−1.71	1593	43	49	19
562	P14618	PKM	Pyruvate kinase isozymes M1/M2	0.003		−1.31	−1.43	1456	41	50	20
799	O00757	FBP2	Fructose-1,6-bisphosphatase isozyme 2	<0.001	1.50	−1.32		919	33	26	9
735	P17174	GOT1	Aspartate aminotransferase, cytoplasmic	0.043		−1.20		839	39	32	13
1486	P05413	FABP3	Fatty acid-binding protein, heart	0.001		1.45	1.29	737	64	23	9
1856	P16219	ACADS	Short-chain specific acyl-CoA dehydrogenase, mitochondrial	0.040		−1.29		325	25	19	8
839	Q13011	ECH1	Delta(3,5)-Delta(2,4)-dienoyl-CoA isomerase, mitochondrial	0.013	1.33		1.42	865	30	22	8
837	Q13011	ECH1	Delta(3,5)-Delta(2,4)-dienoyl-CoA isomerase, mitochondrial	0.017			1.31	622	18	15	6
**Detoxification, cytoprotection**											
885	P07451	CA3	Carbonic anhydrase 3	<0.001	1.46		1.62	754	31	23	6
1821	P07451	CA3	Carbonic anhydrase 3	<0.001	1.23	1.18	1.45	911	45	28	8
1822	P07451	CA3	Carbonic anhydrase 3	<0.001	1.41		1.66	1026	44	31	8
937	P32119	PRDX2	Peroxiredoxin-2	0.003	−1.30			220	24	8	5
928	P04179	SOD2	Superoxide dismutase [Mn], mitochondrial	<0.001		−1.43	−1.56	583	39	21	7
1866	P05091	ALDH2	Aldehyde dehydrogenase, mitochondrial	0.022	1.17			1340	40	48	15
1865	P49189	ALDH9A1	4-trimethylaminobutyraldehyde dehydrogenase	0.012		−1.34		544	26	27	11
815	Q9HC38	GLOD4	Glyoxalase domain-containing protein 4	0.029	1.33		1.39	389	18	10	4
**Proteostasis**											
960	O14558	HSPB6	Heat shock protein beta-6	<0.001	1.21		1.32	763	45	31	6
893	P04792	HSPB1	Heat shock protein beta-1	0.012		1.26	1.25	516	46	17	7
958	P02511	CRYAB	Alpha-crystallin B chain	0.012			1.23	1501	63	76	11
619	P78371	CCT2	T-complex protein 1 subunit beta	0.020	1.18			783	18	20	7
563	P54578	USP14	Ubiquitin carboxyl-terminal hydrolase 14	0.013	−1.25			743	23	18	9
850	P25786	PSMA1	Proteasome subunit alpha type 1	0.012			1.25	430	36	15	7
1854	P28070	PSMB4	Proteasome subunit beta type-4	0.020		1.17		509	33	12	5
**Membrane repair**											
1901	P08758	ANXA5	Annexin A5	0.038	−1.30			888	37	38	11
620	Q6ZMU5	TRIM72	Tripartite motif-containing protein 72	<0.001	1.44		1.29	414	24	8	12
1885	Q6NZ12	PTRF	Polymerase I and transcript release factor	0.022	1.20			432	27	14	8

## Data Availability

All data supporting results are provided in the Tables, Figures, supplementary Figures, supplementary Tables, and in GEO with accession number GSE136344

## Supplementary Materials

The following are available online at https://www.mdpi.com/article/10.3390/ijms22084205/s1, Figure S1: Microarray biological replicates; Figure S2: Correlation between microarray and qRT-PCR; Figure S3: Representative 2DGE and differentially expressed proteins; Figure S4: Correlations between mRNA and protein levels; Table S1: Muscle genes differentially expressed with healthy aging and/or metabolic syndrome in old men; Table S2: Comparison with other transcriptome datasets; Table S3: Over-representation analysis (ORA) for muscle genes up/downregulated with healthy aging; Table S4: Functional class scoring (FCS); Table S5: Positional gene enrichment (PGE) for healthy aging; Table S6: Over-representation analysis (ORA) for muscle genes up/downregulated with metabolic syndrome in old men; Table S7: Positional gene enrichment (PGE) for metabolic syndrome in old men.
